# The Brazilian orthodontic storm

**DOI:** 10.1590/2177-6709.21.3.013-014.edt

**Published:** 2016

**Authors:** David Normando

**Affiliations:** 1 Adjunct professor, Universidade Federal do Pará (UFPA), School of Dentistry, Belém, Pará, Brazil. Coordinator, Graduate program in Dentistry, Universidade Federal do Pará (UFPA) and Specialization course in Orthodontics, ABO-Pará, Belém, Pará, Brazil.

*"Do we have more yesterdays or more tomorrows?"*

*Mia Couto, Mozambican biologist and writer, in "Terra sonâmbula."*

It is not new that Brazilian Orthodontics is praised overseas due to excellent clinical results presented by Brazilian speakers worldwide. What once were intermittent waves breaking in solitude, are now waves that break elsewhere: from Pororoca at Oiapoque to Chuí at Rio Grande; from Maresias in São Paulo to Pipeline in Hawaii. 

Recently, at the American Association of Orthodontics (AAO) annual session held in Orlando, USA, Brazilian speakers stood out. Jorge Faber's speech reminded us of magnificent times when we had Zachrisson & Kokich lecturing. The Brazilian storm also comprehended Lucia Cevidanes, Antonio Carlos Ruellas, Bernardo Souki, Eustáquio Araújo, Leonardo Koerich, Júlio Gurgel, Ana Claudia Conti, Ildeu Andrade, Gustavo Barreto and Nelson Mucha. All of which were incontestable. A number of other Brazilian professors attending the session in Orlando could also have been surfing the event scientific program without running any risk of being swallowed by a wave. 

It all began on our beaches. Clinical quality and valuable communication skills boosted Brazilian participation at our own events. The quality of a Brazilian event used to be determined by the number of foreigner speakers present at that event; however, nowadays, Brazilian speakers' conference rooms are as crowded, or even more crowded, than those of foreigner speakers. The last congress held in Florianópolis (Santa Catarina, Brazil) by the Brazilian Association of Orthodontics (ABOR) was a good example. 

An editorial^1^ previously published in this journal revealed that Brazilian Orthodontics ranks second in scientific production worldwide, according to SCImago. The last issue published by the American Journal of Orthodontics and Dentofacial Orthopedics (AJODO) (May, 2016) is in agreement with such a fact, as half of the ten articles published were written by Brazilian authors. Recent data disclosed by The Angle Orthodontist, as presented by its associate editor Carlos Flores-Mir during AAO 2016, revealed that Brazil is the country with the highest number of manuscripts submitted to the journal, in addition to raking second in the number of publications. Orthodontic science embraces clinical quality.

Nevertheless, clinical excellence together with scientific profuse growth has not kept us away from criticism: "a lot has been produced, but little has been cited." Brazilian Orthodontics ranks second in production, but we remain in sixth when it comes to the number of citations. Although that is a matter of concern for Brazilian dental science as a whole, boosting national orthodontic science impact is the next challenge, and that is a radical maneuver. 

Even though criticism remains sharp, optimism cannot be merely seen as a ripple or wish that tomorrow be postponed. However, there is a need for waves to reach gigantic proportions and, to this end, a sea of enthusiasm must increase in volume... but this has already begun to happen. Take a look. 

The most perceptible fact noticed by those who admire Orthodontics as a science was the number of Brazilian research citations at the AAO meeting. A number of presentations had Brazilian research citations multiplied, as seen *in loco* by DPJO editors. The apex was when American Journal (AJODO), The Angle Orthodontist and European Journal of Orthodontics editors mentioned the quality of Brazilian scientific production. 

We should no longer remain calm on the beach, with our eyes staring at the ocean. Yes, we do have bananas, a storm of radical surfers and an Orthodontics spinning around its own axis on the watch for the perfect wave.
